# Correlates of sedentary time in different age groups: results from a large cross sectional Dutch survey

**DOI:** 10.1186/s12889-016-3769-3

**Published:** 2016-10-26

**Authors:** Claire M. Bernaards, Vincent H. Hildebrandt, Ingrid J. M. Hendriksen

**Affiliations:** 1 TNO (Institute for applied research), P.O. Box 3005, Leiden, 2301 DA The Netherlands; 2 Research Center on Physical Activity, Work and Health, TNO-VU medisch centrum (VUmc), Amsterdam, The Netherlands

**Keywords:** Correlates, Sedentary time, Age groups, Working day, School day, Sedentary work, Physical activity

## Abstract

**Background:**

Evidence shows that prolonged sitting is associated with an increased risk of mortality, independent of physical activity (PA). The aim of the study was to identify correlates of sedentary time (ST) in different age groups and day types (i.e. school-/work day versus non-school-/non-work day).

**Methods:**

The study sample consisted of 1895 Dutch children (4–11 years), 1131 adolescents (12–17 years), 8003 adults (18–64 years) and 1569 elderly (65 years and older) who enrolled in the Dutch continuous national survey ‘Injuries and Physical Activity in the Netherlands’ between 2006 and 2011. Respondents estimated the number of sitting hours during a regular school-/workday and a regular non-school/non-work day. Multiple linear regression analyses on cross-sectional data were used to identify correlates of ST.

**Results:**

Significant positive associations with ST were observed for: higher age (4-to-17-year-olds and elderly), male gender (adults), overweight (children), higher education (adults ≥ 30 years), urban environment (adults), chronic disease (adults ≥ 30 years), sedentary work (adults), not meeting the moderate to vigorous PA (MVPA) guideline (children and adults ≥ 30 years) and not meeting the vigorous PA (VPA) guideline (4-to-17-year-olds). Correlates of ST that significantly differed between day types were working hours and meeting the VPA guideline. More working hours were associated with more ST on school-/work days. In children and adolescents, meeting the VPA guideline was associated with less ST on non-school/non-working days only.

**Conclusions:**

This study provides new insights in the correlates of ST in different age groups and thus possibilities for interventions in these groups. Correlates of ST appear to differ between age groups and to a lesser degree between day types. This implies that interventions to reduce ST should be age specific. Longitudinal studies are needed to draw conclusions on causality of the relationship between identified correlates and ST.

## Background

It is well established that regular engagement in sports activities and moderate to vigorous physical activity (MVPA) have beneficial effects on health outcomes [1, 2]. The importance of sufficient physical activity has reached the general public, not the least by the various promotion campaigns in the last decades. In brief, the message of Dutch campaigns were: being physically active for at least half an hour (adults) or 1 h (children and adolescents) preferably every day, is beneficial for your health. In this message, the remaining 23.5 or 23 h have been left out of consideration. Nowadays, it has become clear that, besides being physically active for 30 to 60 min a day, it is also important to restrict the time spend on sedentary behaviors [3]. Sedentary behavior is characterized by a sitting or supine posture during waking activity accompanied by low caloric energy expenditure [4].

In the last decades, people spend more and more time sitting [5]. In the Netherlands, recent data show that children, adolescents and adults spend on average 7.5, 9.9, and 7.3 h respectively on (self-reported) sedentary activities on a regular school-/working day [6]. Bennie et al. [7] reported that sedentary time (ST) in the Netherlands is the highest in Europe (6.8 versus 5.2 h on average).

Evidence shows that prolonged sitting is associated with an increased risk of mortality and morbidity, independent of physical activity [8, 9] and that prolonged sitting is responsible for nearly 7 % of all-cause mortality [8]. Furthermore, there is evidence for a positive association between sedentary behavior and risk of type 2 diabetes mellitus, cardiovascular disease [10], depression [11] and certain types of cancer [9, 12]. It is therefore necessary that all people, including those who comply with the physical activity guidelines and/or engage in sports activities on a daily basis, pay attention to their sedentary behavior in order to prevent unnecessary health risks.

According to the socio-ecological model, multiple factors that operate at different levels (i.e. individual, social, organizational/community) influence sedentary behaviors [13]. At different ages and different day types (i.e. school-/work days and non-school-/non-work days) people spend time in different social (e.g. students, colleagues, family) and organizational environments (e.g. primary school, secondary school, work) which influences the correlates of sedentary behavior and ST. Since interventions are often age specific it is important to have knowledge on age specific correlates of ST. In addition, this knowledge may also help to identify risk groups. This is one of the first studies that includes almost all age groups, making it possible to compare correlates between age groups because correlates were assessed similarly in all age groups. In contrast to many other studies our large sample size allowed detailed analyses in small age groups. As a result this is one of the first studies that investigated correlates of ST in several relatively small age groups. Furthermore, it is largely unknown whether correlates of ST differ between day types. Previous studies compared ST between week days and weekend days and found higher ST on week days [14, 15]. A disadvantage of working with week days and weekend days is that some people work or go to school during the weekend. Therefore, the aim of this study is to identify correlates of ST in different age groups (children, adolescents, (young) adults, and older adults) and day types.

## Methods

### Study design and data collection

The study sample consisted of Dutch children, adolescents, adults and elderly who enrolled in the Dutch national survey ‘Injuries and Physical Activity in the Netherlands’. This national survey is a continuous cross sectional survey that started in the year 2000 and ended in 2014. Since 2006, data on sedentary behavior have been collected and these data were used in the current study. Between the year 2006 and 2011 approximately 10,000 respondents were questioned each year. Approximately a randomly selected quarter of the total sample was questioned on sport participation and sedentary behavior. Only data of this subgroup could be used to answer our research questions. Data collected between 2012 and 2014 were excluded from the current study due to a modification in the research question about sedentary behavior in the year 2012. Almost all respondents between the age of 4 to 14 years and 65 years and up were interviewed by Computer Aided Telephonic Interviewing (CATI), whereas a mixed mode method was used for respondents in the age group of 15 to 64 years. Mixed mode means that participants could choose whether they were questioned by CATI (40 % of respondents) or whether they filled out an internet-based questionnaire (60 % of respondents). For children (4 to 11 years of age) parents were questioned as a proxy. Adolescents between the age of 12 and 14 were approached via their parents, because the parents needed to give permission for interviewing their children. Older adolescents (15 - to 17 year olds) didn’t need parental permission to participate in the survey. Data of adult participants (18–64 years) were only taken into account if they were students or had a paid job, because only these two groups were questioned about ST on school-/work days. Data of elderly people (65 years and older) were only included if they did not have a paying job because the group of elderly people with a paying job was too small to draw conclusions on.

### Sedentary time

Sedentary time of children on school days was reported by their parents, using the questions ‘Can you estimate the number of hours that your child spends sitting at school on a regular school day, including transport to and from school?’ and ‘Can you estimate the number of hours your child spends sitting or lying after school time on a regular school day, including the evening but excluding sleep time?’. For the older respondents (12 years and up) ST on school-/workdays was estimated by using the questions ‘Can you estimate the number of hours that you spend sitting on a regular school-/workday at school/work, including transport to and from school/work?’ and ‘Can you estimate the number of hours that you spend sitting/lying after school time/work on a regular school-/workday, including the evening but excluding sleep time?’ Total ST at a regular school-/workday was calculated as the sum of ST at school-/work and ST after school/work.

For children (4–11 years) ST on non-school days was reported by their parents, using the questions ‘Can you estimate the number of hours your child spends sitting/lying on a regular non-school day, excluding sleep time (holidays being left out of consideration)?’ For the older respondents (12 years and up), ST on non-school or non-work days was reported using the question ‘Can you estimate the number of hours that you spend sitting/lying on a regular non-school/non-work day, excluding sleep time (holidays being left out of consideration)?’.

### Correlates of sedentary time

#### Socio-demographic variables

The following variables were assessed: age (years), gender, educational level of the person with the highest income in the household (Lower education: no school graduation/lower general secondary education; Middle education: higher general secondary education/pre-university education/vocational school; High education: Bachelor or Master degree), having sedentary work (yes/no), working hours per week and the level of urbanity (non-urban, slightly urban, moderately urban, highly urban and very highly urban). Urbanity was estimated by postal code and expressed as the number of postal addresses per square kilometer. Respondents having sedentary work were those who indicated to mainly sit at work (Question: Do you mainly walk, stand or sit at work?).

#### Health and behavioral variables

Physical activity was assessed using physical activity questions that were specially developed for the continuous national survey in order to estimate the proportion of respondents meeting the Dutch physical activity guidelines (Appendix). Both face validity (in adults) [16] and criterion validity (in youth) of these questions have been studied [17]. The Dutch moderate to vigorous physical activity (MVPA) guideline focuses on the long-term maintenance of health and is different for youth and adults (18 years and older). Children (4–17 years) meet the MVPA guideline if they engage in MVPA during at least 60 min a day at all days of the week, during summer- and wintertime (i.e. meet the MVPA guideline in summer and winter). Adults meet the MVPA guideline if they engage in MVPA during at least 30 min a day at minimally five days a week, during summer- and wintertime. Examples of MVPA are activities such as walking, cycling, gardening, sports or exercise at work or at school, e.g. all activities with intensities that are at least equal to walking at a firm pace or cycling [18]. In addition, it was assessed whether respondents met the Dutch vigorous intensity physical activity (VPA) guideline (Appendix). This guideline focuses on the maintenance of aerobic fitness. People of all age groups meet this guideline if they engage in VPA during at least 20 min at least three times a week, during summer- and wintertime. VPA is defined as strenuous physical/sport activity during leisure time that makes you sweat [18]. Respondents were asked whether they engaged in sports activities during the past 12 months and if they did so, how many times a week or how many times a year. Respondents were considered to be a sports participant if they engaged in sports activities at least once a week or at least 40 times a year, regardless of the type of sport [19]. Finally, respondents were asked to report their body weight (in kilograms) and body height (in meters) and whether or not they suffered from one or more chronic diseases. Body mass index (BMI) was calculated according to the formula BMI = body height/(body weight)^2^. Age dependent cut off points for BMI were used in order to categorize children and adolescents into weight categories: underweight, normal weight, and overweight [20, 21]. Body weight of adults and elderly was categorized into underweight (BMI < 20 kg/m^2^), normal weight (BMI 20–25 kg/m^2^) and overweight (BMI ≥ 25 kg/m^2^).

### Statistics

All analyses were conducted using SPSS for Windows, version 20.0. Descriptive subject characteristics were presented as mean values (± SD) and percentages. To examine the association between potential correlates of ST (independent variables) on the one hand and ST (dependent variable) on the other hand, multiple linear regression analysis was conducted. All independent variables were put into the model at once. All independent variables that were significantly associated with ST after correction for all other independent variables, were called correlates. For all age groups the model included socio-demographic, health and behavioral variables, i.e. gender, age, weight category (underweight or normal weight versus overweight), urbanity (non-urban versus slightly urban, moderately urban, highly urban or very highly urban), chronic disease (one or more chronic diseases versus no chronic disease), educational level of the person with the highest income in the household (low education versus middle education and high education), meeting the MVPA guideline (yes/no), meeting the VPA guideline (yes/no), sport participation (sports participant versus non-sports participant). For adults the variables sedentary work (sedentary work versus non-sedentary work) and working hours a week were additionally included to the model. Dummy variables were used for urbanity and educational level of the person with the highest income in the household.

All covariates included in the model were tested for multicollinearity. No correlation coefficients above 0.8 between all pairs of the independent covariates were observed [22]. Separate analyses were run for ST during regular school-/workdays and during non-school/non-work days. A probability value of *P* < 0.05 was considered significant. Differences in ST between age groups were tested with ANOVA and by using dummy variables for age groups.

## Results

### Participant characteristics

Participant characteristics are presented in Table 1. Of all adults who participated in the study between 2006 and 2011, and answered questions about sedentary behavior, 20.6 % (2074 out of 10077) were excluded for not having a paid job or not being a student. The final study sample consisted of 1895 Dutch children (4–11 years), 1131 adolescents (12–17 years), 8003 adults (18–64 years) and 1569 elderly (65 years and older). On both day types ST differed significantly between age groups (*p* < 0,01). ST in children was significantly lower than in all older age groups. Reported differences in ST were small between adolescents, 18–29-year-olds, 30–49-year-olds, and 50–64-year-olds and often not statistically significant. Most of the respondents were living in highly urban areas.

**Table 1:** Characteristics of participants

	4–11 years		12–17 years		18–29 years		30–49 years		50–64 years		≥ 65 years	
	N	% or mean (SD)	N	% or mean (SD)	N	% or mean (SD)	N	% or mean (SD)	N	% or mean (SD)	N	% or mean (SD)
Questionnaire	1895		1131		1938		4263		1802		1569	
Online			336	29.7	1757	90.7	3515	82.5	1411	78.3	21	1.3
Telephone		100.0	795	70.3	181	9.3	748	17.5	391	21.7	1548	98.7
Gender (%)	1895		1131		1938		4263		1802		1569	
Male	990	52.2	541	47.8	858	44.3	2348	55.1	1065	59.1	638	40.7
Female	905	47.8	590	52.2	1080	55.7	1915	44.9	737	40.9	931	59.3
Age (years)	1895	7.6 (2.3)	1131	14.2 (1.7)	1938	23.6 (3.5)	4263	39.5 (5.6)	1802	55.2 (3.8)	1569	72.6 (5.9)
Weight category (%)^a^	1579		1036		1885		4106		1766		1514	
Underweight	362	22.9	121	11.7	349	18.5	215	5.2	45	2.5	57	3.8
Normal weight	957	60.6	815	78.7	1069	56.7	1736	42.3	624	35.3	610	40.3
Overweight	260	16.5	100	9.7	467	24.8	2155	52.5	1097	62.1	847	55.9
Urbanity (%) ^b^	1802		1084		1922		4215		1775		1448	
Non-urban	319	17.7	190	17.5	188	9.8	477	11.3	222	12.5	197	13.6
Slightly urban	471	26.1	262	24.2	353	18.4	856	20.3	364	20.5	331	22.9
Moderately urban	388	21.5	256	23.6	380	19.8	892	21.2	394	22.2	309	21.3
Highly urban	420	23.3	266	24.5	597	31.1	1281	30.4	505	28.5	387	26.7
Very highly urban	204	11.3	110	10.1	404	21.0	709	16.8	291	16.4	224	15.5
One or more chronic diseases (%)	1895	9.0	1131	12.2	1938	13.7	4263	20.8	1802	28.0	1569	34.0
Education level breadwinner (%)^c^	1858		834		1726		4093		1742		1526	
Low	319	17.2	163	19.5	204	11.8	646	15.8	462	26.5	850	55.7
Middle	799	43.0	342	41.0	597	34.6	1752	42.8	652	37.4	385	25.2
High	740	39.8	329	39.4	925	53.6	1695	41.4	628	36.1	291	19.1
Sedentary work (%)	N/A	N/A	N/A	N/A	1606	47.3	4126	58.4	1731	54.8	N/A	N/A
Working hours/week	N/A	N/A	N/A	N/A	1638	30.1 (14.3)	4248	35.1 (11.9)	1801	33.5 (12.5)	N/A	N/A
Sedentary time school/workday (hours/day)	1886	6.3 (2.6)	1122	9.3 (3.3)	1881	9.7 (4.3)	4152	9.4 (4.7)	1700	9.4 (4.7)	N/A	N/A
Sedentary time non-school/non-work day (hours/day)	1756	3.8 (2.2)	1005	5.6 (3.4)	1804	5.8 (3.1)	3939	5.5 (3.2)	1658	5.8 (3.2)	1569	4.6 (2.9)
Meeting the MVPA guideline (%)^d^	1811	24.7	997	13.8	1938	44.1	4263	44.9	1802	48.7	1569	54.9
Meeting the VPA guideline (%)^e^	1777	33.0	1043	37.9	1816	25.1	4015	20.8	1711	19.2	1494	10.8
Sports participant (%)^f^	1895	68.2	1131	74.4	1938	61.0	4263	52.1	1802	52.8	1569	37.3

^a^ Age dependent cut off points for body mass index (BMI) were used in order to categorize children and adolescents into the weight categories: underweight, normal weight, and overweight. Body weight of adults and elderly was categorized into underweight (BMI < 20 kg/m^2^), normal weight (BMI 20–25 kg/m^2^) and overweight (BMI ≥ 25 kg/m^2^)

^b^ Based on postal codes

^c^ Lower education: no school graduation/lower general secondary education; Middle education: higher general secondary education/pre-university education/vocational school; High education: Bachelor or Master degree

^d^ Children (4–17 years) meet the moderate to vigorous intensity physical activity (MVPA) guideline if they engage in MVPA during at least 60 min a day at all days of the week, during summer- and wintertime. Adults and elderly meet the MVPA guideline if they engage in MVPA during at least 30 min a day at minimally five days a week, during summer- and wintertime

^e^ People of all age groups meet this guideline if they engage in vigorous intensity physical activity (VPA) during at least 20 min at least three times a week, during summer- and wintertime

^e^ Sports participant: Engaging in sports activities at least once a week or at least 40 times a year, regardless of the type of sport

*N/A* not applicable

### Correlates of sedentary time

Table 2 and 3 present the significant associations between socio-demographic, health and behavioral variables and ST on school-/workdays (Table 2) and non-school/non-work days (Table 3) for each age group. All associations were adjusted for the other variables in the model. The variables associated with ST on school-/workdays differed between age groups. Table 4 summarizes the findings from Table 2 and 3 and shows which variables were positively, negatively or not associated with ST on school-/workdays and non-school/non-work days for each age group.

**Table 2:** Regression coefficients and 95 % confidence intervals of significant associations between socio-demographic, health and behavioral variables (independent variables) and sedentary time on school-/work days (dependent variable)

Age	b	95 % CI
4–11 years^a^		
Age	0.40	[0.34; 0.46]^**^
Overweight^c^	0.69	[0.35; 1.02]^**^
Meeting the MVPA guideline^d^	−0.34	[−0.63;−0.05]^*^
12–17 years^a^		
Age	0.45	[0.30; 0.59]^**^
18–29 years^b^		
Gender ^e^	−0.48	[−0.91;−0.05]^*^
Age	−0.11	[−0.18;−0.04]^**^
Overweight	0.55	[0.07; 1.04]^*^
Urbanity ^f^		
Slightly urban	0.20	[−0.62; 1.02]
Moderately urban	0.73	[−0.09; 1.54]
Highly urban	1.06	[0.28; 1.84]**
Very highly urban	1.02	[0.17; 1.87]*
Sedentary work ^g^	3.99	[3.51; 4.43]^**^
30–49 years^b^		
Gender	−0.84	[−1.16;−0.53]^**^
Overweight	0.29	[0.03; 0.55]^*^
Urbanity		
Slightly urban	−0.03	[−0.50; 0.45]
Moderately urban	0.34	[−0.13; 0.81]
Highly urban	0.44	[−0.00; 0.89]
Very highly urban	0.89	[0.39; 1.39]**
Educational level breadwinner^h^		
Middle educational level	0.23	[−0.17; 0.62]
High educational level	0.55	[0.13; 0.96]**
Sedentary work	4.37	[4.09; 4.65]^**^
Working hours/week	0.03	[0.01; 0.04]^**^
Meeting the MVPA guideline	−0.94	[−1.21;−0.67]**
Sports participation ^i^	−0.29	[−0.56;−0.02]*
50–64 years^b^		
Urbanity		
Slightly urban	0.51	[−0,25; 1.27]
Moderately urban	0.99	[0.25; 1.74]**
Highly urban	0.55	[−0.17; 1.27]
Very urban	0.79	[−0.00; 1.59]
Chronic disease ^j^	0.55	[0.08; 1.01]*
Educational level breadwinner		
Middle educational level	0.58	[0.04; 1.12]*
High educational level	0.37	[−0.19; 0.93]
Sedentary work	4.32	[3.87; 4.77]^*^
Working hours/week	0.03	[0.01; 0.05]^**^
Meeting the MVPA guideline	−0.75	[−1.19;−0.31]^**^
Sports participation	−0.67	[−1.10;−0.28]^**^

^a^ Associations were corrected for gender, age, weight category, urbanity, chronic disease, education level breadwinner, meeting the moderate to vigorous intensity physical activity (MVPA) guideline, meeting the vigorous intensity physical activity (VPA) guideline, sports participation

^b^ Associations were corrected for gender, age, weight category, urbanity, chronic disease, education level breadwinner, sedentary work, working hours/week, meeting the MVPA guideline, meeting the VPA guideline, sports participation

^c ‘^Overweight’ regressed against ‘Underweight or normal weight’ (reference)

^d^ ‘Meeting the moderate to vigorous intensity physical activity (MVPA) guideline’ regressed against ‘Not meeting the MVPA guideline’(reference)

^e^ ‘Female’ regressed against ‘Male’ (reference)

^f ‘^Slightly urban environment’, ‘moderately urban environment’,’highly urban environment’ and ‘very highly urban environment’ regressed against non-urban environment (reference)

^g‘^Sedentary work’ regressed against ‘Non-sedentary work’ (reference)

^h^ ‘Middle education’ and ‘High education’ regressed against ‘Low education’ (reference)

^i^ ‘Sports participant’ regressed against ‘Non-sports participant’ (reference)

^j^ ‘Having one or more chronic diseases’ regressed against ‘Not having a chronic disease’ (reference)

^*^ *P* < 0.05

^**^ *P* < 0.01

**Table 3:** Regression coefficients and 95 % confidence intervals of significant associations between socio-demographic, health and behavioral variables (independent variables) and sedentary time on non-school-/non-work days (dependent variable)

Age	b	95 % CI
4–11 years^a^		
Age	0.24	[0.18; 0.29]^**^
Overweight ^c^	0.36	[0.06; 0.66]^*^
Meeting the MVPA guideline ^d^	−0.45	[−0.72;−0.19]^**^
Meeting the VPA guideline ^e^	−0.35	[−0.59;−0.11]^**^
12–17 years^a^		
Age	0.31	[0.14; 0.47]^**^
Meeting the VPA guideline	−0.66	[−1.14;−0.09]^*^
18–29 years^b^		
Gender ^f^	−0.41	[−0.74;−0.06]^*^
Age	−0.09	[−0.15;−0.03]^**^
Sedentary work ^g^	0.50	[0.13; 0.87]^**^
30–49 years^b^		
Gender	−0.45	[−0.71;−0.19]^**^
Overweight	0.39	[0.18; 0.60]^**^
Urbanity ^h^		
Slightly urban	−0.29	[−0.68; 0.10]
Moderately urban	0.01	[−0.37; 0.40]
Highly urban	0.40	[0.03; 0.76]*
Very highly urban	0.38	[−0.03; 0.78]**
Chronic disease ^i^	0.62	[0.36; 0.88]^**^
Sedentary work	0.97	[0.74; 1.20]^**^
Meeting the MVPA guideline	−0.37	[−0.60;−0.15]^**^
Sports participation ^j^	−0.38	[−0.61;−0.16]^**^
50–64 years^b^		
Gender	−0.59	[−0.98;−0.20]^**^
Urbanity		
Slightly urban	0.36	[−0.25; 0.97]
Moderately urban	0.37	[−0.23; 0.96]
Highly urban	0.32	[−0.26; 0.89]]
Very highly urban	0.70	[0.06; 1.33]*
Chronic disease	0.60	[0.20; 0.96]^***^
Educational level breadwinner ^k^		
Middle educational level	0.11	[−0.33; 0.55]
High educational level	0.50	[0.05; 0.95*]
Sedentary work	0.81	[0.45; 1.17]^**^
Working hours/week	−0.02	[−0.03;−0.00]^*^
Sports participation	−0.61	[−0.96;−0.25**]
≥ 65 years^a^		
Age	0.06	[0.03; 0.09]^**^
Chronic disease	0.84	[0.52; 1.16]^**^
Educational level breadwinner		
Middle educational level	0.27	[−0.10; 0.63]
High educational level	0.44	[0.03; 0.85]^*^
Meeting the MVPA guideline	−0.86	[−1.17;−0.55]^**^

^a^ Associations were corrected for gender, age, weight category, urbanity, chronic disease, education level breadwinner, meeting the moderate to vigorous intensity physical activity (MVPA) guideline, meeting the vigorous intensity physical activity (VPA) guideline, sports participation

^b^ Associations were corrected for gender, age, weight category, urbanity, chronic disease, education level breadwinner, sedentary work, working hours/week, meeting the MVPA guideline, meeting the VPA guideline, sports participation

^c ‘^Overweight’ regressed against ‘Underweight or normal weight’ (reference)

^d^ ‘Meeting the moderate to vigorous intensity physical activity (MVPA) guideline’ regressed against ‘Not meeting the MVPA guideline’(reference)

^e^ ‘Meeting the vigorous intensity physical activity (VPA) guideline’ regressed against ‘Not meeting the VPA guideline’ (reference)

^f^ ‘Female’ regressed against ‘Male’ (reference)

^g^ ‘Sedentary work’ regressed against ‘Non-sedentary work’ (reference)

^h^^‘^Slightly urban environment’, ‘moderately urban environment’,’highly urban environment’ and ‘very highly urban environment’ regressed against non-urban environment (reference)

^i^‘Having one or more chronic diseases’ regressed against ‘Not having a chronic disease’ (reference)

^j^ ‘Sports participant’ regressed against ‘Non-sports participant’ (reference)

^k^ ‘Middle education’ and ‘High education’ regressed against ‘Low education’ (reference)

*P < 0.05

^**^ *P* < 0.01

**Table 4:** Type of association for each correlate of sedentary time; + positive association, - negative association, 0 no association

Correlates	Age and day type										
	4–11		12–17		18–29		30–49		50–64		65 years and older
	School	Non school	School/work	Non school/non work	School/work	Non school/non Work	School/work	Non school/non work	School/Work	Non school/non work	Non school/Non work
Age	+	+	+	+	-	-	0	0	0	0	+
Female gender	0	0	0	0	-	-	-	-	0	-	0
Educational level breadwinner	0	0	0	0	0	0	+	0	+	+	+
Urbanity	0	0	0	0	+	0	+	+	+	+	0
Overweight	+	+	0	0	+	0	+	+	0	0	0
Chronic disease	0	0	0	0	0	0	0	+	+	+	+
Sedentary work	Not applicable	Not applicable	0	0	+	+	+	+	+	+	Not applicable
Working hours/week	Not applicable	Not applicable	0	0	0	0	+	0	+	-	Not applicable
Meeting the moderate to vigorous intensity physical activity (MVPA) guideline	-	-	0	0	0	0	-	-	-	0	-
Meeting the vigorous intensity physical activity (VPA) guideline	0	-	0	-	0	0	0	0	0	0	0
Sports participation	0	0	0	0	0	0	-	-	-	-	0

#### 4–11-year-olds

In children, both higher age and overweight were associated with more ST on both day types (i.e. school days and non-school days). Meeting the MVPA guideline was associated with less ST on both day types, whereas meeting the VPA guideline was associated with less ST on non-school days only.

#### 12–17-year-olds

In adolescents, higher age was associated with more ST on both day types, which means that older adolescents are more sedentary than younger adolescents. Meeting the VPA guideline was associated with less ST on non-school days, similar as in children.

#### 18–29-year-olds

In young adults, higher age and female gender were associated with less ST, whereas sedentary work was associated with more ST on both day types. Furthermore, on school-/workdays, a higher level of urbanity and overweight were associated with more ST.

#### 30–49-year-olds

In this age group, female gender, meeting the MVPA guideline, and sports participation were associated with less ST on both day types. A higher level of urbanity, overweight and sedentary work were all associated with more ST on both day types. Furthermore, a higher educational level of the person with the highest income in the household and more working hours a week were both associated with more ST on school-/workdays. Having a chronic disease was associated with more ST on non-school/non-work days.

#### 50–64-year-olds

On both day types, a higher educational level of the person with the highest income in the household, a higher level of urbanity, having a chronic disease and sedentary work were associated with more ST, whereas sports participation was associated with less ST. Meeting the MVPA guideline was associated with less ST on school-/workdays. Number of working hours showed conflicting results: whereas more working hours were associated with more ST on school-/workdays, the opposite association was found on non-school/non-work days in 50-to-64-year-olds. Finally, female gender was associated with less ST on non-school/non-work days.

#### Elderly (65-years and older)

In the oldest age group, no data were collected about school-/workdays. On non-school/non-work days, meeting the MVPA guideline was associated with less ST. Older age, higher education of the person with the highest income in the household and having a chronic disease were associated with more ST.

## Discussion

The aim of this study was to study the correlates of ST in different age groups and day types. Correlates that were consistently associated with higher ST were higher age (in 4-to-17 year olds and respondents of 65 years and older), higher educational level (in respondents of 50 years and older), higher urbanity (in respondents of 30 years and older), overweight (in 4-to-11-year olds and 30-to-49-year olds), chronic disease (in respondents of 30 years and older), sedentary work (in 18-to-64-year olds) and more working hours on school-/workdays (in 30-to-64 year olds).

Correlates that were consistently associated with less ST were female gender (in 18-to-49 year olds), meeting the MVPA guideline (in 4-to-11 year olds, 30-to-49 year olds and respondents of 65 years and older), meeting the VPA guideline (in 4-to-17 year olds; non-school/non-work day only) and sports participation (in 30-to-64 year olds).

### Correlates of sedentary time

#### Age

Our finding that ST increases with age in children and adolescents is in line with prior studies [23–27]. Two prospective studies using accelerometry showed that sedentary behavior increased with age, at the expense of light physical activity [25] and MVPA [23]. Higher ST was also observed in older adolescents compared with younger adolescents in a cross sectional study using accelerometry [24]. A cross sectional study that used self-report showed that higher age among adolescents was associated with more leisure computer use but not with more television viewing [26]. Among adults, the results with regard to age as a correlate of ST are mixed and seem dependent on the type of sedentary behavior (e.g. computer use versus television viewing) [28]. This dependency on type of sedentary behavior might explain why age was not a consistent correlate of ST (among adults) in the current study, because only total ST was assessed. In elderly, higher age is often associated with more ST [29] similar as in the current study. The results of this study show that older adolescents (as compared with younger adolescents) and older elderly (as compared to younger elderly) are risk groups for spending much time on sedentary behaviors. Although significant differences were found in ST between age groups we cannot draw conclusions on changes in ST across age groups based on our cross sectional data.

#### Gender

In the current study, gender was not a correlate of ST among children and adolescents similar as reported by Stierlin et al. [27]. Stierlin et al. [27] found no evidence for an association between gender and subjectively measured sedentary behavior in youth and inconsistent evidence for the association between gender and screen time. Other studies showed mixed results. In one cross-sectional study [24] and one longitudinal study [25], adolescent boys spent less time on sedentary behaviors than adolescent girls, but the differences were small. In two large cross sectional studies boys reported more screen time than girls [26, 30], but in one of these studies girls reported more ST [30]. In the current study we concluded that women reported less ST than men, which is in line with recently published correlates of sitting in European adults [7]. European men reported more ST (320 min) than women (301 min) on a mean weekday, whereas other studies showed mixed results [28, 31]. Bauman et al. [31] found higher levels of ST among men in seven out of 20 countries worldwide, and higher levels of ST among women in five out of 20 countries with the remainder showing no differences. In their systematic review, Rhodes et al. [28] concluded that gender may not affect sedentary behaviors, with the exception of video games which are played more frequently by men than by women. In the current study we were unable to discriminate between different types of sedentary behavior. Therefore it is unknown whether the observed gender differences in ST can be attributed to differences in gaming time. These findings suggest that adult men are at increased risk of spending more time on sedentary activities.

#### Educational level

The current study suggests that higher education is associated with more ST in adults (≥30 years) on school-/work days. Only in 50-to-64-year-olds this positive association was found on non-school/non-work days as well. A review study showed that the relationship between education and ST is dependent on the type of sedentary behavior; more TV viewing hours in lower educated people and more computer hours in higher educated people [28]. Whether the positive association between educational level and ST in the current study results from more computer hours or other sedentary activities is unknown because different types of sedentary behavior were not assessed.

#### Sedentary work

Having a sedentary job was the strongest correlate of ST in adults. On school-/work days, ST in sedentary workers was about 4 h a day higher than in non-sedentary workers, and 30 to 60 min higher on non-school/non-work days. Since having a sedentary job was associated with more ST on both day types, it seems that adults with sedentary work were unable to compensate their sedentary work with non-sedentary activities during leisure time. This finding is in line with the findings of Saidj et al. [32] who reported more ST outside of work in workers with sedentary occupations on both working and non-working days. Workers with sedentary jobs in the study of Saidj et al. [32] reported less TV/DVD time but more leisure sitting and non-screen sitting time, which suggests that adults with sedentary jobs prefer other types of sedentary behavior than adults without sedentary jobs. Our findings are not in line with two other studies [33, 34] that reported no differences in leisure time sitting between workers with sedentary jobs (‘white collar workers’) and workers with less sedentary jobs (‘blue collar workers’) on working days. Tudor-Locke et al. [35] even reported opposite results compared to the results of the current study; i.e. workers in higher level of intensity-defined occupations spend more time on sedentary behaviors outside of work than workers in sedentary occupations. The observed differences between the current study and other studies [32–35] might be explained by differences in the way total ST was measured and the types of sedentary behavior that were included to calculate total ST. Furthermore, most studies investigated ST on working days when people spend time on other sedentary behaviors than on non-working days [32]. Based on the results of the current study we conclude that interventions to reduce ST in adults should primarily focus on ways to make the work less sedentary.

#### Working hours

More working hours a week were associated with more ST in 30–64-year-olds on school-/work days similar as in other studies [34, 36, 37]. In the current study, this positive association was not found on non-school/non-working days. In 50-to-64-year olds, on the other hand, more working hours were associated with less ST on non-school/non-work days, which suggests that older workers with full-time jobs seem to compensate high ST on school-/work days with less ST on non-school/non-work days. This compensation effect was not found by Clemes et al. [37] who found more ST in full-time workers compared to part-time workers on both working days and non-working days.

#### Urbanity

An urban environment was independently associated with more ST in adults (18–64 years) but not in children, adolescents and elderly. In 30-to-64-year-olds this association was found on both day types. The association between urban environment and more ST in adults was also found in other studies [36, 38, 39]. Our results are not in line with the results from a recently published cross sectional study conducted in five European countries [40]. In this study total ST did not differ between high- and low-density neighborhoods despite differences in total moderate to vigorous physical activity levels for transport. Since it is unknown what causes higher ST in adults living in urban environments, more research is needed to find out how urban environments may contribute to higher sedentary time.

#### Sports participation and physical activity

Although several studies have investigated the association between physical activity and ST [28], to our knowledge, this is one of the first studies that explores the association between sports participation and ST. A cohort study showed smaller increases in children’s ST after school in children who played sport as a family compared to children who did not [41]. The results of the current study suggest that the association between physical activity and ST is dependent on the type of physical activity (moderate, vigorous, or sports activity) and the age of the respondents. Whereas higher sports participation was associated with lower ST in adults, it was not associated with ST in children and adolescents. Meeting the MVPA guideline, on the other hand, was associated with less sedentariness in both children and adults, but not in adolescents. These findings are more or less in line with the findings of Leech et al. [42], who showed that clustering of sedentary behavior and physical activity differs according to age, gender and socio-economic status. Moreover, there are studies showing that the association between physical activity and ST is dependent on the type of sedentary behavior. Rhodes et al. [28] concluded that higher physical activity levels seem to be associated with less television viewing and general screen viewing but not with computer use or other sedentary behavior.

#### Weight

In the current study, we found mixed results with regard to the association between overweight and ST. Overweight was associated with more ST in children, 18-to-29-year-olds (only on school-/work days) and 30-to-49-year-olds, but not in other age groups. Although several studies reported a significant association between overweight and higher self-reported ST in children [30, 43], studies among adults showed mixed results with regard to the association between BMI and ST. In their systematic review, Rhodes et al. [28] concluded that there is some evidence for a relationship between BMI and TV viewing/general screen time, but the relationship between BMI and other sedentary behaviors did not seem to be strong.

#### Chronic disease

In the older age groups (≥ 30 years), having a chronic disease was associated with more ST. This association has also been reported in a cross-sectional study among middle-aged Australian males [44]. It seems plausible that adults with one or more chronic diseases are more likely to experience difficulties with physical activities and are more likely to be sedentary than adults without chronic diseases.

### Methodology

The strength of this study is primarily the availability of data on ST on both school-/work days and non-school-/non-work days, a large sample size and respondents of all age groups, which allowed detailed analyses in small age groups. Knowledge on age- and day type specific correlates of ST is important for the development of interventions and the identification of risk groups. In addition, we were able to study the independent association between different types of physical activity (MVPA, VPA and sports participation) and ST. By using continuous national survey data we were able to study the association with ST and many relevant variables. However, as our data come from an existing dataset we could only include variables in the model that were available in the dataset. Therefore, we were unable to analyse all possible relevant correlates of ST. Another limitation comes from the cross-sectional design of the study, implicating that no conclusions about causality can be drawn. Also, all data were obtained by parent- or self-report which increases that risk of (recall) bias and social desirability bias. Little is known on the validity of parent-reported ST of children. A recent study found no correlation between parent-reported and accelerometer measured ST of young children (age < 6 years) [45]. Sarker et al. [45] asked parents to record how often their children engaged in selected sedentary behaviors such as television viewing or playing on the computer. In the present study, parents were asked to report the total ST of their children, which might be even more difficult to estimate. As far as we know, the validity of the latter method is unknown. However, in absence of a valid method we used this proxy-method and recognize that validation of this way of measuring ST in children is needed. Furthermore, we were unable to discriminate between different types of sedentary behavior (e.g. television viewing and computer use) or different settings during school-/work days and non-school-/non-work days (e.g. transport, school, work, leisure time). Several studies have shown that correlates of ST may differ between types of sedentary behavior [26, 28, 46] and settings [32]. As a result, correlates of sedentary behavior that are highly setting specific or sedentary behavior type specific could not be identified.

## Conclusions

The results of this study show that correlates of ST differed largely between age groups and less between day types. In youth, more ST was associated with higher age, overweight, and not complying with the physical activity guidelines. In adulthood, sedentary work was the strongest correlate of ST and even associated with more ST on non-school/non-work days. In addition, female gender, urbanity and sports participation were correlates of ST in adults but not in children, adolescents and older adults. In young adults correlates differed slightly from correlates in the other adult age groups, for instance with regard to sports participation and age. Due to the cross sectional design of our study we cannot draw conclusion on causality. The results of the current study give some indications for intervention developers but longitudinal studies are needed to explore the causality of the significant associations between correlates and ST. Based on the current study we can identify risk groups for high ST within age groups. These are for instance overweight children and children who don’t meet the physical activity guidelines (within age group 4-to-11-year-olds), older adolescents (within age group 12–17-year-olds), adults with sedentary work, male adults, adults (aged 30–64) who don’t participate in sports and live in urban areas (within age group 18–64-year-olds), and higher age-groups in the 65+ group.

## Appendix

Questions from the Dutch monitor ‘Injuries and Physical Activity in the Netherlands’ to calculate the proportion of Dutch children, adolescents and adults that meet the MVPA and VPA guidelines.

### MVPA guideline

For the questions below, please think about physical activities such as walking, cycling, gardening, sports or exercise at work or at school. This involves all activities of which the intensity is at least equal to walking at a firm pace or cycling.

Adults:

1. On how many days a week do you engage in such activities for at least 30 min a day during the summer?
  *Please report the average number of days for a regular week. If it is less than one*, *please report zero*.
  □ Days a week
2. And on how many days a week do you engage in such activities for at least 30 min a day during the winter?
  *Please report the average number of days for a regular week. If it is less than one*, *please report zero*.
  □ Days a week

Children/adolescents:

3. And on how many days a week do you engage in such activities for at least 60 min a day during the summer?
  *Please report the average number of days for a regular week. If it is less than one*, *please report zero*.
  □ Days a week
4. And on how many days a week do you engage in such activities for at least 60 min a day during the winter?
  *Please report the average number of days for a regular week. If it is less than one day a week*, *please report zero*.
  □ Days a week

### VPA guidelines

All age groups:

The following questions are about vigorous intensity physical activity during leisure time.

5. On how many times a week during the summer do you engage in sports or other vigorous intensity physical activities in such a way that it makes you sweat?
  *Please*, *only report episodes with a minimum of 20 min. If it is less than once a week*, *please report zero*.
  □ Times a week
6. On how many times a week during the winter do you engage in sports or other vigorous intensity physical activities in such a way that it makes you sweat?
  *Please*, *only report episodes with a minimum of 20 min. If it is less than once a week*, *please report zero*.
  □ Times a week

## Abbreviations

BMI: Body mass index
CATI: Computer Aided Telephonic Interviewing
MVPA: Moderate to Vigorous intensity Physical Activity
VPA: Vigorous intensity Physical Activity
